# Assessment of Land-Use Impact on Macroinvertebrate Communities in the Zwalm River Basin (Flanders, Belgium) Using Multivariate Analysis and Geographic Information Systems

**DOI:** 10.1100/tsw.2002.111

**Published:** 2002-03-02

**Authors:** Veronique Adriaenssens, Peter L. M. Goethals, Niels De Pauw

**Affiliations:** Laboratory of Environmental Toxicology and Aquatic Ecology, Ghent University, J. Plateaustraat 22, B-9000 Gent, Belgium

**Keywords:** environmental impact assessment, biological indicators, Geographic Information System, multivariate analysis, macroinvertebrates

## Abstract

Relationships between land-use and river water quality assessed by means of biological and physical-chemical variables and habitat characteristics were analysed for the Zwalm River basin in Flanders (Belgium). The research focussed on three zones within this river basin, each characterized by different land uses, and consequently, different types of pollution, mainly of diffuse origin. Environmental data have been integrated within a Geographic Information System. Possible relationships between aquatic ecosystem and land-use variables were searched for by means of multivariate analysis.

## INTRODUCTION

Despite significant efforts during the last decade to improve water quality in Flanders (Belgium), aquatic communities in rivers still remain in poor condition. Although point source discharges have been reduced, most streams are still characterised by bad or moderate water quality, primarily because of nonpoint source pollution. Among nonpoint sources, agricultural disturbances carry a significant risk to stream communities. Diffuse pollution varies spatially and temporally across a catchment in response to a combination of land conditions and topographic factors[1]. In Flanders, increased activities in agricultural regions together with a higher demand for water supplies have resulted in a growing concern about water quality. A high degree of spatial dispersion of housing also is a major problem in relation to water quality that probably can be solved only by introducing small-scale wastewater treatment plants.

To assess these disturbances of agricultural and domestic origin, macroinvertebrate community composition can be used. Macroinvertebrate taxa are important ecological indicators for the assessment of the ecological integrity of a river basin and can be used in river basin management. Studies have revealed that biological impacts from nonpoint sources and habitat degradation may not be fully demonstrated by periodic measurements of the physical-chemical characteristics of water bodies[2]. Biological assessments are necessary in this context to reveal long-term and mixed-stressor impacts on the aquatic biological communities[3]. Biological indices can be used to assess the effect of land use, although it is not possible to detect specific cause-effect relationships without controlled experimental research. However, by means of multivariate techniques, contemporary environmental data can be used to explain biological variation[4,5] and can reveal likely relationships between land-use changes and macroinvertebrate taxa. Then potential biological indicators can be assigned.

Spatial scale of impact assessment on the aquatic ecosystem is of major importance. Site-specific environmental assessments that fail to account for the larger-scale, cumulative, basin-wide consequences of catchment alteration (by anthropogenic or natural processes) will not accurately characterise biotic responses to environmental changes[6,7,8,9]. Because of this need, many land management and regulatory agencies are now promoting "watershed analysis" or "ecosystem analysis at the watershed scale" as an appropriate methodology for characterizing aquatic ecosystem conditions in land-use planning[10,11]. Using a river catchment as a unit for integrated land-use assessment and management presents a number of methodological and technical challenges that exemplify many of the key issues involved in linking Geographic Information Systems (GIS) with environmental models for decision support[12].

River basin management in Flanders is based on site-specific assessments of physical-chemical characteristics and monitoring of macroinvertebrate communities. Because of the important contribution of nonpoint sources of pollution in Flanders and the complexity of the impacts, it is difficult to identify potential relationships in the river basins. For management and especially land-use planning, insight into these specific cause-effect relationships is necessary. In this way, one could demonstrate which indicators are important for site-specific assessments as well as assessments at catchment scale.

The aim of this article is to assign possible ecological indicators based on data of macroinvertebrate communities and environmental variables that can explain possible relationships of land-use activities in the Zwalm River basin (Fig. 1). GIS was used for impact zone delineation. By means of the Belgian Biotic Index (BBI), a general assessment of the sampling zones within the Zwalm River basin was made. Multivariate analysis was conducted on the data set and evaluated for use in river basin assessment and management.

## METHODS

### Study Area

The Zwalm River basin (Fig. 1) is part of the Upper-Scheldt River basin and consists mainly of small brooks. It has a total surface of 11,650 ha. The Zwalm River itself has a length of 22 km and is characterised by a very irregular flow regime. The water quality in the Zwalm River basin substantially improved in the last 2 years, due to investments in sewer systems and wastewater treatment plants. Nevertheless, untreated urban wastewater and diffuse pollution originating from agricultural activities still significantly impact it. The Zwalm Valley has a landscape with several height differences. The fauna in the upper parts is unique and is characterised by the presence of the Bullhead (*Cottus gobio*) and the Brook Lamprey (*Lampetra planeri*) and several sensitive and vulnerable macroinvertebrates (e.g., mayflies taxa Heptageniidae)[13].

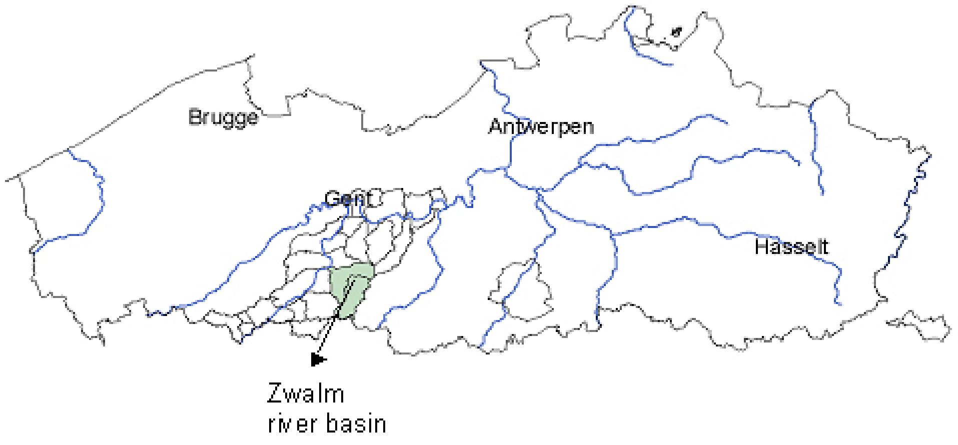

**FIGURE 1.** The Zwalm River basin in Flanders (Belgium).

Eight measuring sites were selected within three subbasins in the Zwalm River basin (Fig. 2). Each subbasin is subjected to a different kind of land-use and, as a consequence, different types of impacts and eventual pollution. The first study area (Sassegembeek) is situated near one of the springs in the woods of Brakel and can be considered as a reference zone (i.e., hardly affected by any source of pollution). The second one (Wijlegembeek) is part of an agricultural area with diffuse pollution. The third one (Passemarebeek) is affected by domestic as well as agricultural pollution. These sites were sampled during five monitoring campaigns from February 2001 until June 2001.

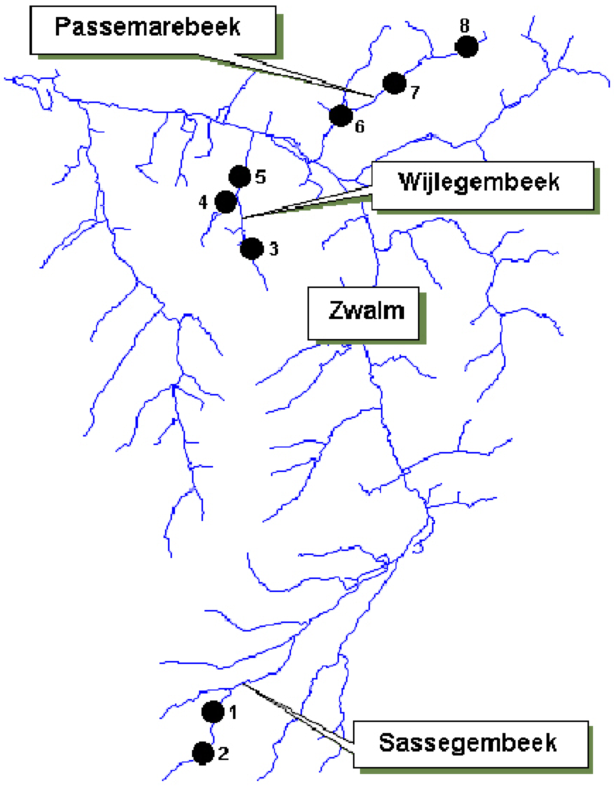

**FIGURE 2**. Sampling sites in the Zwalm River basin.

## Biological, Habitat, Land-Use, and Physical-Chemical Monitoring

The macroinvertebrates were collected by means of kick sampling with a standard hand net (mesh size = 500 µm)[14]. The objective of the sampling consists of collecting the most representative diversity of macroinvertebrates at the station examined. The sampling effort covered a 10-min sampling time, exploring all accessible aquatic habitats or microbiotopes[15]. The assessment of the water quality was based on the BBI. This biological assessment method uses macroinvertebrates as indicators of the level of pollution and is a standardised method in Belgium[14]. The scoring system ranges from 10 to 0, where 9–10 = unpolluted, 7–8 = slightly polluted, 5–6 = moderately polluted, 3–4 = heavily polluted, 1–2 = very heavily polluted, and 0 = dead. The scoring system is based on the theorem that increasing pollution will result in a loss of diversity and a progressive elimination of certain pollution-sensitive groups[15,16].

The biological variables consist of all macroinvertebrate taxa (Table 1) that were identified at the levels as specified by the BBI[14]. The measured physical-chemical, habitat, and land-use variables are shown in Table 2.

**TABLE 1**
**Macroinvertebrate Taxa, Taxonomic Groups, and Abbreviations**

| ***Taxonomic Group*** | **Abbreviation** | **Maxcroinvertebrate Taxon** |
|---|---|---|
| Oligochaeta | Lumu | Lumbriculidae |
| | Tubi | Tubificidae |
| Hirudinea | Erpo | *Erpobdella* |
| | Glosi | *Glossiphonia* |
| | Ther | *Theromyzon* |
| Mollusca | Gyra | *Gyraulus* |
| | Lymn | *Lymnaea* |
| | Myx | *Myxas glutinosa* |
| Crustacea | Asel | Asellidae |
| | Gamm | Gammaridae |
| Ephemeroptera | Baet | *Baetis* |
| | Epher | *Ephemera* |
| | Hept | *Heptagenia* |
| Odonata | Corde | *Cordulegaster* |
| Plecoptera | Bracyp | Brachyptera |
| Megaloptera | Sial | *Sialis* |
| Coleoptera | Dryo | Dryopidae |
| | Dyti | Dytiscidae |
| | Hydran | Hydraenidae |
| | Hydroph | Hydrophilidae |
| | Note | Noteridae |
| | Scir | Scirtidae |
| Trichoptera | Limne | Limnephilidae |
| | Phry | Phryganeidae |
| Diptera | Chirthu | Chironomidae *thummi-plumosus* |
| | Chirnon | Chironomidae *non thummi-plumosus* |
| | Ptyc | Ptychopteridae |
| | Scio | Sciomyzidae |
| | Simu | Simuliidae |
| | Taba | Tabanidae |
| | Tipu | Tipulidae |

**TABLE 2**
**Physical-Chemical, Habitat, and Land-Use Variables**

| Variables | Measuring Units |
|---|---|
| Temperature | °C |
| pH | - log [H+] |
| Conductivity | µS/cm |
| Suspended solids | mg/l |
| Dissolved oxygen | mg/l |
| Water level | cm |
| Fraction pebbles | % surface |
| Fraction sand | % surface |
| Shadow | % surface |
| Aquatic macrophytes | Presence/absence |
| Width | cm |
| Stream velocity | m/s |
| Meandering | 6 categories: 1 (well developed) to 6 (absent) |
| Hollow banks | 6 categories: 1 (well developed) to 6 (absent) |
| Pools/riffles | 6 categories: 1 (well developed) to 6 (absent) |
| Artificial embankment | Presence/absence |
| Total area land-use upstream of sampling site | $10^4 m^2$ |
| Agricultural area upstream of sampling site | $10^4 m^2$ |
| Housings area upstream of sampling site | $10^4 m^2$ |
| Forest area upstream of sampling site | $10^4 m^2$ |

Land-use data of the Zwalm River basin at scale 1/100,000 were obtained from the Flemish Land Agency and originated from the EU CORINE programme. The electronic hydrological data were based on the Flemish Hydrographical Atlas. Using ArcView GIS 3.2[19], overlays and analyses were made. Buffer zones along the watercourses were defined by means of the X-tools extension[20] of ArcView GIS 3.2. Subbasin delineation was done with the Basin extension[21] within the Spatial Analyst program of ArcView GIS 3.2.

The collected data were analysed by multivariate analysis[17] in PC-ORD 3.20 program[18]. To reduce the dominating influence of abundant taxa in the multivariate analysis, abundance data of macroinvertebrates were log (n + 1) transformed[4,22,23]. Because of the skewed distribution of the physical-chemical variables, log transformation was applied to become a log normal distribution[5,23,24] and land-use variables were log (n + 1) transformed to be appropriate for use in multivariate analysis.

## RESULTS AND DISCUSSION

### Delineation of Impact Zones with GIS

The first step of the land-use impact evaluation was the delineation of the impact zones on the sampling points within the Zwalm River basin based on GIS. The two methods, buffer zone delineation and subbasin delineation, resulted in different input values for the land-use variables (Table 1) in the consequent multivariate analysis. The first option was to define buffer zones along the watercourses. A 100-m buffer zone was aligned at each site of the watercourses, and the land-use area was calculated from the source up to the sampling point (Fig. 3). The implementation of this technique was rather easy, but seemed to be inconvenient when surface runoff is an important impact factor, which is the case for the southern area of the Zwalm River basin. Therefore, a second technique to select a watershed within a larger catchment was used, which is called subbasin delineation (Fig. 3).

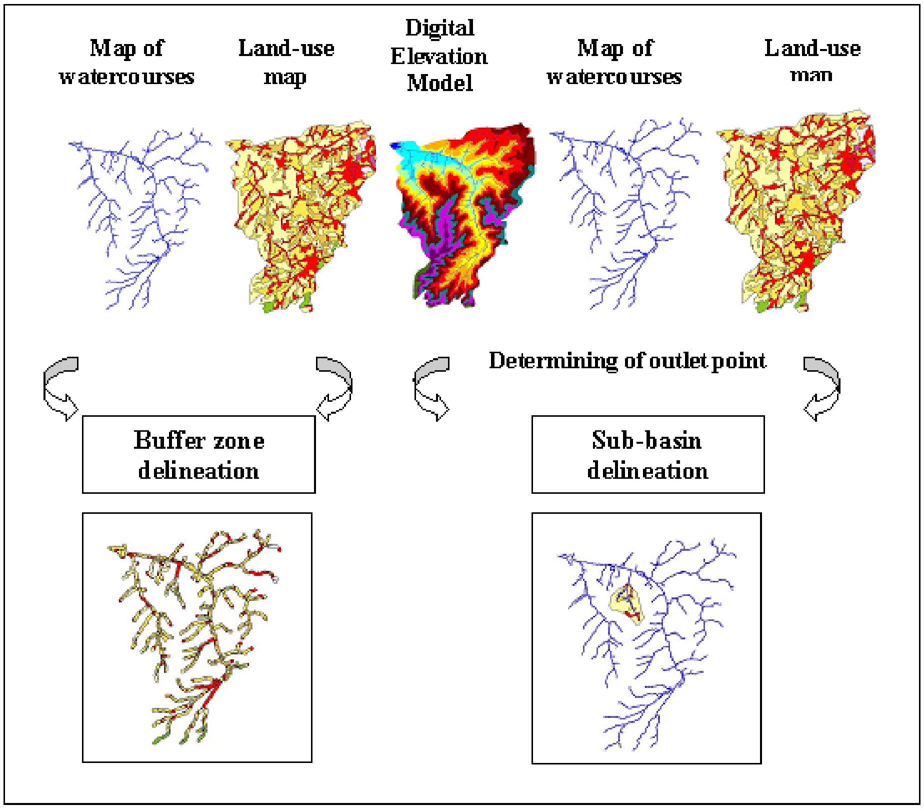

**FIGURE 3**. Delineation of impact zones with a Geographic Information System. Left: Buffer zone delineation with X-tools in ArcView GIS 3.1, based on the land-use map and map of watercourses. Right: Subbasin delineation with Basin in Spatial Analyst ArcView GIS 3.1, based on the digital elevation model, land-use map, and map of watercourses.

The area of interest for the analysis is selected by picking a point (the sampling point) on the terrain, and the application automatically computes the contributing area that makes up the local watershed. Besides being a useful way to query a catchment, this procedure enforces an important constraint. Using this technique, the watershed is always defined with a single outlet point. Analysis ensures all water flows to a single low point along the watershed boundary[1]. This delineation of impact zones takes height differences and, consequently, runoff into account and thus requires a detailed digital elevation map. Because of the lack of detail of the obtained digital elevation map of the Zwalm River basin, the buffer zone delineation was used for impact zone delineation.

The impact of diffuse pollution on the aquatic ecosystem originating from agricultural activities will be further analysed by means of a detailed digital elevation map and soil data of the Zwalm basin. The combination of soils that are not covered with vegetation and steeper slopes may contribute to high sediment loads in the receiving streams. This may harm the stream habitat by loss of pool depth and increased turbidity and is also the cause of an important part of nutrient runoff. Also, effluent discharge points and vegetation strips along the watercourses need to be included in future analyses. The interdependence of land-use, riparian buffers, and river morphology is a complicated issue. All these characteristics determine the structure of the aquatic macroinvertebrate communities. The implementation of these characteristics in the analysis will probably give a better approximation of the water flow from agricultural areas and the effective domestic pollution on the aquatic ecosystem.

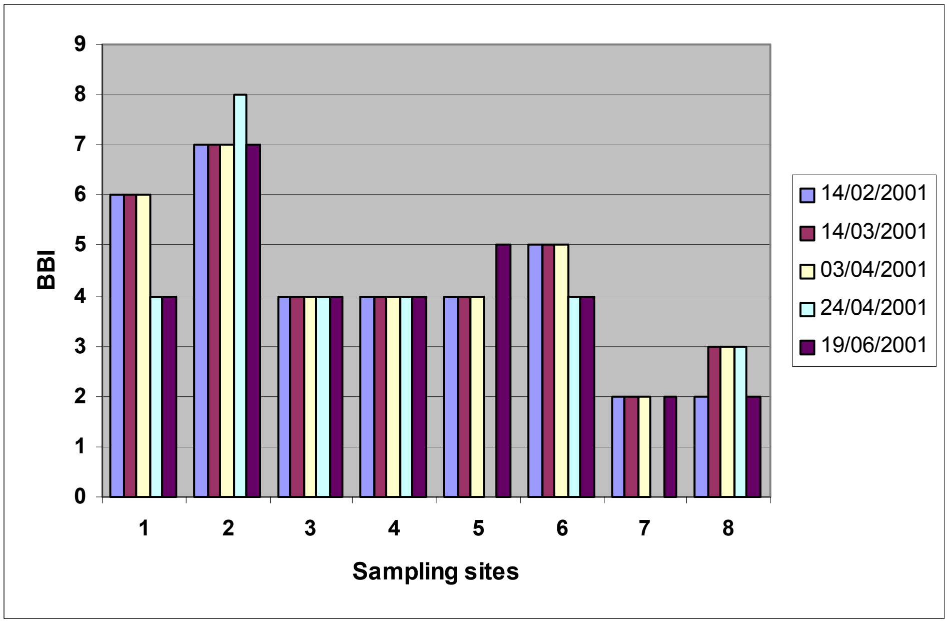

**FIGURE 4**. Biological assessment of sampling sites within the Zwalm River basin by means of the BBI.

## Analysis of Land-Use Impacts on Aquatic Macroinvertebrate Communities

Analysis of land-use impacts on aquatic macroinvertebrate communities was based on biotic indices and multivariate analysis. The BBI, based on the total number of taxa present as well as the presence of particular indicator groups, was used to assess the water quality at the different sampling sites. In Fig. 4, the evolution of the BBI in time and space is presented. Sites 1 and 2 of the forest zone have the highest BBI values; however, only sampling site 2 in the Sassegembeek, which is the site nearest to the source, met the good water-quality standard of BBI $\geq$ 7. The Wijlegembeek (sites 3, 4, and 5) in the agricultural zone was assessed as heavily polluted, except for two sampling periods at point 5. For the Passemarebeek (sites 6, 7, and 8), subjected to mixed household and agricultural impacts, the upstream sampling sites 7 and 8 had a BBI of only 2 or 3, illustrating the impact of domestic pollution from the nearby village. These sites had a worse quality than the downstream site 6 because of the possible dilution effect of the confluence with another brook just before sampling point 6.

Although simple metrics can be more easily understood than multivariate statistics, they have several limitations. The consequence of an increasing amount of pollution is mainly the disappearance of sensitive species, so the presence/absence of the nontolerant taxon gives enough information about the state of the aquatic ecosystem and the consequences on the taxon and can be detected by the BBI. In the range of bad to heavily and very heavily polluted streams, it will not be easy to detect improvements or deteriorations of water quality because the sensitive species have already disappeared and because a change in macroinvertebrate communities will rather be detected by a change in abundance of the remaining taxa. Also, small changes in habitat characteristics of the river can be as important as pollution in determining the distribution of many taxa and will not be detected by means of this biotic index. Management of watercourses related to land-use planning needs more specific indicators to assess and predict biological community responses. Much of the information about a community is lost when combined in one integrated index. Therefore, multivariate statistics have become very useful in the analysis of biological monitoring data[11], and knowledge can be extracted from abundance data of macroinvertebrates.

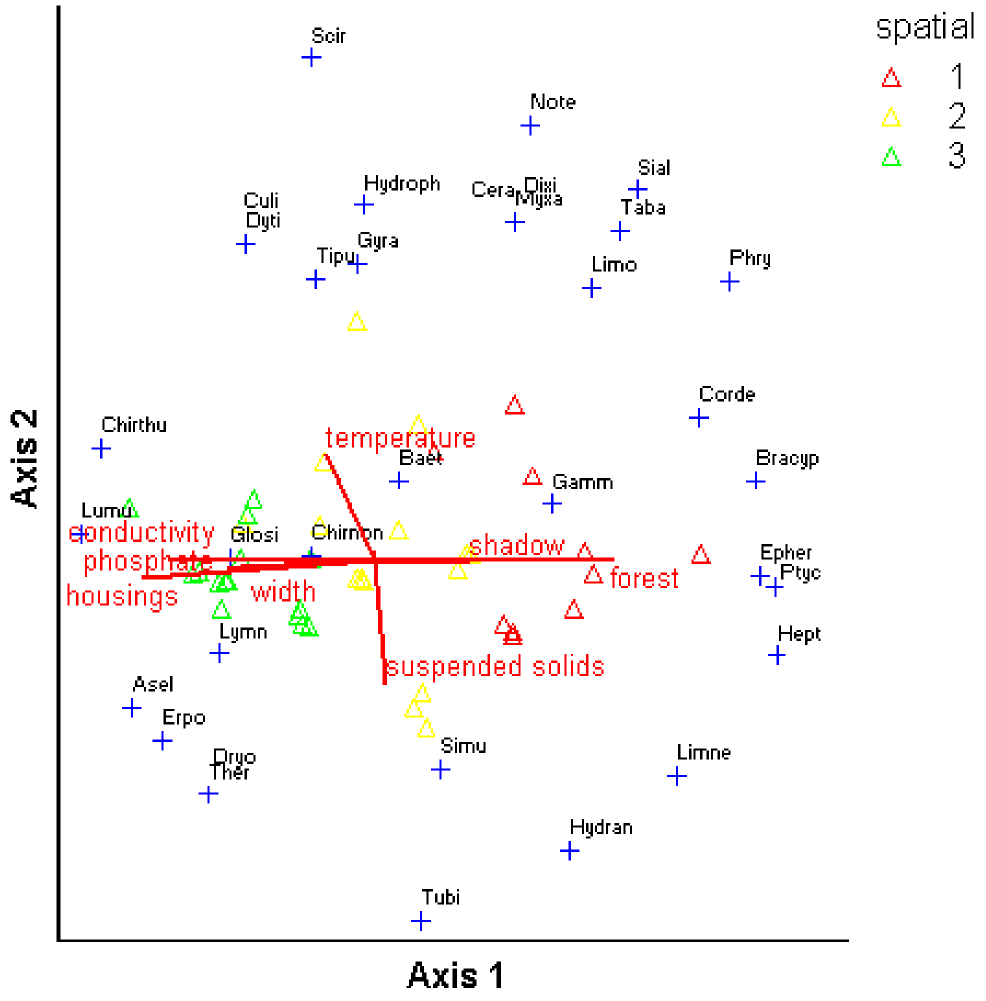

**FIGURE 5**. DCA biplot for log abundance of macroinvertebrate taxa and log-transformed environmental indicators. Spatial 1 (red): Sassegembeek sampling sites. Spatial 2 (yellow): Wijlegembeek sampling sites. Spatial 3 (green): Passemarebeek sampling sites.

Indirect and direct gradient analyses were made on the data set of macroinvertebrate data and environmental variables (Table 2) by means of detrended correspondence analysis (DCA) and canonical correspondence analysis (CCA). The length of gradient of the first DCA axis confirmed that the macroinvertebrate data were suitable for unimodal analysis by DCA and CCA[25]. The indirect gradient analysis revealed that the measured environmental variables sufficiently predict the main part of the variation in the macroinvertebrate taxa composition, if they strongly relate to the first few ordination axes[23]. In direct gradient analysis, species occurrences are related directly to environmental variables[26]. In CCA, species are assumed to have Gaussian (bell-shaped) response surfaces with respect to compound environmental gradients. These gradients are assumed to be linear combinations of the environmental variables[23].

The DCA biplot (Fig. 5), which is the result of gradient analysis on macroinvertebrate data, shows that sites from the three sampling zones at the different sampling periods can be clearly separated in the ordination space. This allows us to recognise the different impacts of land-use activities (forest, agriculture, and domestic) on the aquatic ecosystem based on only macroinvertebrate data. Therefore, one can conclude that different land-use activities are clearly reflected in the composition of aquatic macroinvertebrate communities. In contrast to the BBI assessment, nearly all sites are classified as heavily polluted, and no specific relationship could be highlighted.

The DCA biplot revealed a strong correlation between phosphate, housing, and conductivity. Also, the variables of forest and shadow were strongly correlated. Phosphate, housing, and conductivity are strongly negatively correlated with forest and shadow variables. From these correlations, one might conclude that domestic perturbations are reflected mainly in physical-chemical pollution, while agricultural activities have an impact on mainly the habitat quality. The concentration of suspended solids seemed to be an important factor in explaining the macroinvertebrate community structure and is indeed an extremely important problem in the monitored region because of the important height differences, the removal of natural banks, and intensive agriculture activities along the watercourses. Temporal dependency was included into the multivariate analysis, but was not observed to be as important as was reported by Ruse[4].

From the DCA, Ephemeroptera taxa, *Heptagenia* and *Ephemera*, Plecoptera taxa, *Brachyptera*, and Trichoptera taxa, Phryganeidae and Limnephilidae, are grouped together and show a strong correlation with the land-use variable of forest area and shadow. These groups are already implemented into the EPT (Ephemeroptera, Plecoptera, and Trichoptera) index, but cannot be used for assessment for land-use impact in Flanders at the moment because of the rarity of the EPT taxa. Chironomidae *non-thumi*, Lumbricidae, and *Glossiphonia* act as important indicators for domestic land-use impact. Simulidae were significant for detecting the impact of suspended solids.

But, it is nontrivial to detect by indirect gradient analysis the effects on community composition of a subset of environmental variables in which one is particularly interested[27]. These limitations can be overcome only by methods of direct gradient analysis as a CCA for unimodal analysis, in which species occurrences are related directly to environmental variables[26]. From the CCA data (Fig. 6), it can be seen that the difference with the indirect gradient analysis (Fig. 5) for the plots of macroinvertebrate taxa is quite high.

Ter Braak[23] stresses that CCA can be used in combination with DCA, but when the solutions differ, then the environmental variables account for less conspicuous directions of variation in the species data or that environmental variables cannot account for any of the variation. This is the case for the Zwalm data, where the first two axes in a CCA with log-transformed abundance data of macroinvertebrates and log-transformed environmental variables account for only 26.1% variance in the data set (Table 3). One may suggest that the known environmental variation accounts for just a very small part of the community variation. This makes a reliable interpretation of the gradient analysis more difficult.

When different sets of environmental variables are used for the direct gradient analysis (Table 3), the distribution of the taxa can be analysed along each land-use, habitat, or physical-chemical variable in the different CCA outputs. Because these variables do not relate strongly to the first few axes, they cannot account for the main part of the variation. The analysis also reveals that land-use and habitat characteristics seem to be more important for the structure of the macroinvertebrate communities in the Zwalm River basin than physical-chemical variables, even when assuming a large nutrient input from agricultural and domestic sites.

Specific land-use biological indicators for agricultural or housing pollution, on the other hand, were difficult to assign based on direct gradient analysis because of the low percentage of variance explained. Land-use impacts also have, aside input of pollutants to the environment, an important impact on structural characteristics of watercourses, and this should also be taken into account in river management.

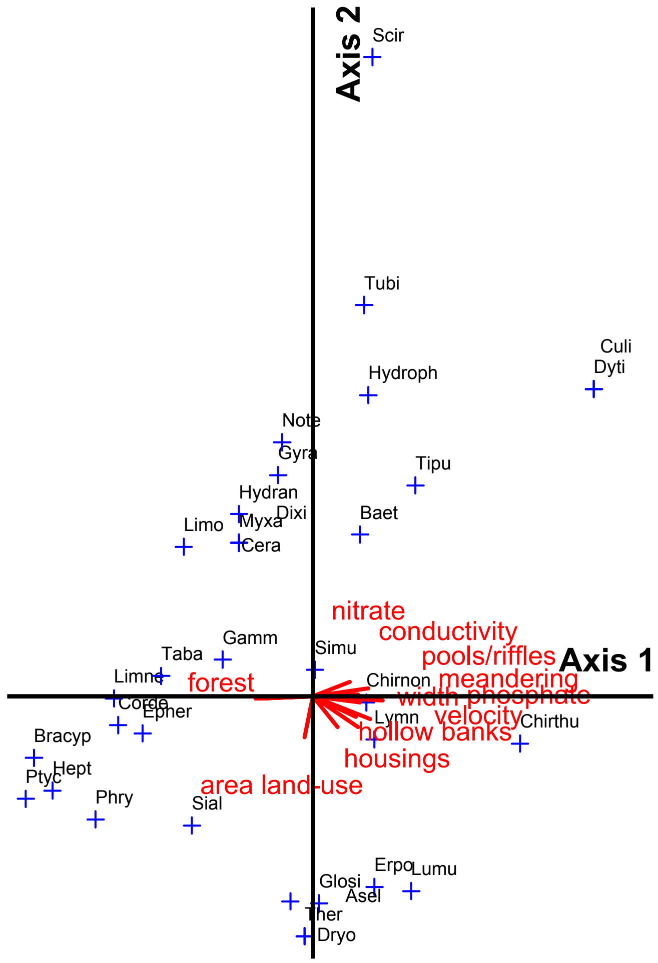

**FIGURE 6**. CCA biplot for log abundance of macroinvertebrate taxa and log-transformed environmental indicators.

**TABLE 3**
**CCA Analysis Results: Percentage Variance Explained by the First Two Axes for the Macroinvertebrate Data Set and Different Sets of Environmental Variables**

| CCA % Variance Explained | Axis 1 | Axis 2 | Axis 1 + Axis 2 |
|---|---|---|---|
| Log abundance/ log environmental variables | 16.2% | 9.9% | 26.1% |
| Log abundance/ log land use | 14.2% | 8.2% | 22.4% |
| Log abundance/ log habitat | 16.2% | 5.2% | 21.4% |
| Log abundance/ log physical-chemical variables | 13.3% | 5.5% | 18.8% |

## FUTURE PROSPECTS

This article presents results from preliminary research that focuses on scaling related to impact zones, variable selection, and analysis techniques such as multivariate analysis. It is obvious from the results that land-use impact assessment implies a multidisciplinary approach to implement all the influencing factors concerning the impacts on the aquatic ecosystem. In the future, a framework for river basins has to be developed to predict where land-use disturbances hold a significant risk for aquatic communities. Therefore, it must be suitable to link specific stressors to responses in aquatic communities. Other analysis techniques such as artificial intelligence will be used for prediction of macroinvertebrate responses on impact factors[28], and the monitoring network will be enlarged to get a more robust data set.

## CONCLUSIONS

Nonpoint source pollution, mainly of agricultural and domestic origin, causes important water-quality problems in Flanders. Therefore, in river basin management, specific biological indicators are needed to relate specific causes of land-use activities to aquatic biological community changes. Monitoring of macroinvertebrate communities and physical-chemical, land-use, and habitat characteristics in three zones within the Zwalm River basin, each characterised by different land uses, has been done. GIS is a very appropriate tool to combine all river basin information. This allows a transparent land-use impact assessment on the headwaters and streams and improves the delineation of the impact zone. The explanatory value of multivariate analysis can be of great importance when searching for indicator species that can account for specific land-use impacts, but the results of the direct gradient analysis are often difficult to interpret. From the indirect gradient analysis, specific responses of macroinvertebrate communities to the different land-use activities became obvious. Land-use activities appeared to have more effect on macroinvertebrate communities by changing habitat characteristics than by physical-chemical properties, and this should be taken into account when developing ecological indicators.

## ACKNOWLEDGEMENTS

The first author is a recipient of a grant from the Institute for the Promotion of Innovation by Science and Technology in Flanders (IWT).

## References

1. Pullar, D., and Springer, D. (2000) Towards integrating GIS and catchment models. *Environ. Model. Software* **15**, 451–459.
2. Wang, X. (2001) Integrating water-quality management and land-use planning in a watershed context. *J. Environ. Manage.* **61**, 25–36.
3. Karr, J.R. and Dudley, D.R. (1981) Ecological perspectives on water quality goals. *Environ. Manage.* **5**, 55–68.
4. Ruse, L.P. (1996) Multivariate techniques relating macroinvertebrate and environmental data from a river catchment. *Water Res.* **30(12)**, 3017–3024.
5. Bis, B., Zdanowicz, A., and Zalewsi, M. (2000) Effects of catchment properties on hydrochemistry, habitat complexity and invertebrate community structure in a lowland river. *Hydrobiologia* **422/423**, 369–387.
6. Fore, S., Karr, J.R., and Wisseman, R.W. (1996) Assessing invertebrate responses to human activities evaluating alternative approaches. *J. North Am. Benthol. Soc.* **15**, 212–233.
7. Roth, N.E., Allan, J.D., and Erickson, D.L. (1996) Landscape influences on stream biotic integrity assessed at multiple spatial scales. *Landscape Ecol.* **11**, 141–156.
8. Richards, C., Johson, L.B., and Host, G.E. (1996) Landscape-scale influences on stream habitats. *Can. J. Fish. Aquat. Sci.* **53**, 295–311.

9. Allan, J.D., Erickson, D.L., and Fay, J. (1997) The influence of catchment land use on stream integrity across multiple spatial scales. *Freshwater Biol.* **37**, 149–162.
10. Jensen, M.E., Reynolds, K., Andreasen, J., and Goodman, I. (2000) A knowledge-based approach to the assessment of watershed condition. *Environ. Monit. Assess.* **64**, 271–283.
11. Watzin, M.C. and McIntosh, A.W. (1999) Aquatic ecosystems in agricultural landscapes: a review of ecological indicators and achievable ecological indicators. *J. Soil Water Conserv.* **54(4)**, 636–644.
12. Aspinall, R. and Pearson, D. (2000) Integrated geographical assessment of environmental condition in water catchments: linking landscape ecology, environmental modelling and GIS. *J. Environ. Manage.* **59,** 299–319.
13. Goethals, P. and De Pauw, N. (2001) Development of a concept for integrated river assessment in Flanders, Belgium. *J. Limnol*., in press.
14. NBN (1984) Qualité biologique des cours d'eau: détermination de l'indice biotique se basant sur les macro-invertebres aquatiques, NBN T92-402. Institut Belge de Normalisation (IBN).
15. De Pauw, N. and Vanhooren, G. (1983) Method for biological quality assessment of watercourses in Belgium. *Hydrobiologia* **100**, 153–168.
16. De Pauw, N. and Vannevel, R. (1991) Macroinvertebrates and water quality. Dossiers Stichting Leefmilieu, Antwerpen. Dossier No. 11. 316 p. (in Dutch).
17. Jongman, R.H.G., Ter Braak, C.J.F., and van Tongeren, O.F.R. (1987) Data analysis in community and landscape ecology, Wageningen, the Netherlands, 299 p.
18. McCune, B. and Mefford, M.J. (1997) *Multivariate Analysis of Ecological Data Version 3.20.* MJM Software, Gleneden Beach, OR.
19. ArcView GIS 3.2.a. Environmental Research Institute, Inc.
20. De Laune, M. (2000) The X-tools extension. http://gis.esri.com/arcscripts/scripts.cfm.
21. Petras, I. (2000) Basin. http://gis.esri.com/arcscripts/scripts.cfm.
22. Thorne, R.S.J., Williams, W.P., and Cao, Y. (1999) The influence of data transformations on biological monitoring studies using macroinvertebrates. *Water Res*. **33(2)**, 343–350.
23. Ter Braak, C.J.F. (1986) Canonical correspondence analysis: a new eigenvector technique for multivariate direct gradient analysis. *Ecology* **67(5)**, 1167–1179.
24. Delong, M.D. and Brusven, M.A. (1998) Macroinvertebrate community structure along the longitudinal gradient of an agriculturally impacted stream. *Environ. Manage.* **22(3)**, 445–457.
25. Ter Braak, C.J.F. and Prentice, I.C. (1988) A theory of gradient analysis. *Adv. Ecol. Res.* **18**, 271–317.
26. Gauch, H.G. (1982) Multivariate analysis in community ecology. Cambridge University Press, Cambridge, England.
27. Carleton, T.J. (1984) Residual ordination analysis: a method for exploring vegetation-environment relationships. *Ecology* **65**, 469–477.
28. Goethals, P., Dzeroski, S., Dedecker, A., Raes, N., Adriaenssens, V., Gabriels, W., and De Pauw, N. (2001) Comparing classification trees, artificial neural networks and fuzzy logic models to predict macroinvertebrate communities in the Zwalm river basin (Flanders, Belgium). Abstracts book, p. 66. 2nd Symposium for European Freshwater Sciences, Toulouse, France.

## BIOSKETCH

**Veronique Adriaenssens** is a Ph.D. student in Agricultural and Applied Biological Sciences at Ghent University (Belgium). She is the recipient of a grant of the Institute for the Promotion of Innovation by Science and Technology in Flanders (IWT).
